# Impacts of anaesthesia strategies on mouth–lung microbial signature: Evidence from bronchoscopy sampling and sequencing

**DOI:** 10.1002/ctm2.1355

**Published:** 2023-07-28

**Authors:** Chunli Tang, Chenting Zhang, Qian Jiang, Rongmei Geng, Jingnan Zhai, Jian Wang, Shiyue Li, Kai Yang

**Affiliations:** ^1^ State Key Laboratory of Respiratory Disease National Clinical Research Center for Respiratory Disease, Guangzhou Institute of Respiratory Health, The First Affiliated Hospital of Guangzhou Medical University Guangzhou Guangdong P. R. China; ^2^ Guangzhou National Laboratory, Guangzhou International Bio Island Guangzhou Guangdong P. R. China; ^3^ Section of Physiology, Division of Pulmonary, Critical Care and Sleep Medicine, Department of Medicine University of California San Diego La Jolla California USA

Dear Editor,

Bronchoscopy has become a well‐established strategy for the diagnosis and treatment of lung diseases and is commonly used in pathological assessment and biopsy. Bronchoscopy can be operated under either general or local anaesthesia, considering procedural purpose, anaesthetic tolerance, adequate patient comfort and side‐effects.^1^, ^2^, ^3^ Studies have reported fever and bacterial infection in cases post‐bronchoscopy assessment,^4^, ^5^, ^6^ implying the possibility of microbial contamination during the procedure. Given that bronchoscopy was proven to be a reliable operation that merely affected the lung microbial community,^7^ this study first compared the effects of two commonly used anaesthesia strategies on lung microecosystem by using bronchoscopy sampling and sequencing. Although general anaesthesia slightly enhanced mouth–lung microbial communication, likely through increased aspiration, similar mouth–lung microbial diversity and composition were observed under general and local anaesthesia strategies.

Qualified participants (n = 59) were enrolled by screening adult patients with asymptomatic pulmonary nodules, those were scheduled for transbronchial lung biopsy from January 2021 to November 2021 at the First Affiliated Hospital of Guangzhou Medical University after exclusion of subjects with signs of infectious lung diseases and recent usage of antibiotics and/or steroids (Figure 1A), and assigned into general anaesthesia (n = 28) and local anaesthesia (n = 31) groups following strategies detailed in the Supporting Information, with matched age, sex and smoking status (Figure 1B). Before the bronchoscopy operation, all participants were mouth rinsed with sterile saline (SS) and sampled as the oral rinse specimen (OR). Bronchoalveolar lavage (BAL) samples were collected in left lingular segment (L) and right middle lung lobe (R), which can also be defined as a healthy lung lobe without a pulmonary nodule (H) and a nodular lung lobe (N), by using SS rinsed through a sterile brush (Figure 1C). Pure SS, brush saline and scope saline rinse were used as negative controls. The OR and BAL samples were quality controlled, amplified, sequenced and analysed (Supporting Information Figures S2 & S3).

**FIGURE 1:**
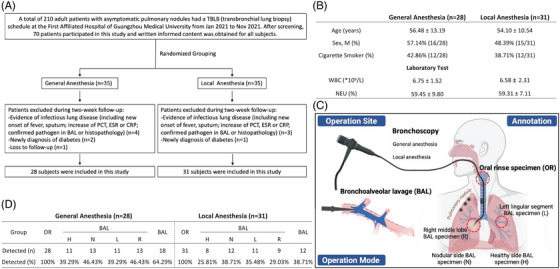
Study design and recruitment strategy. (A) Flow chart showing the inclusion and exclusion protocols for the subjects involved in this study. (B) Demographic and laboratory features of subjects. Data were presented as mean ± SD or percentage. WBC, White blood cells; NEU, neutrophile. (C) Graphic illustration of the operation site, mode and annotation. (D) Table showing the microbial detection number and rate in samples of oral rinse specimen (OR) and bronchoalveolar lavage specimen (BAL), as well as different BAL subgroups, including left lingular segment lung lobe specimen (L), right middle lung lobe specimen (R), healthy lung lobe specimen (H) and nodular lung lobe specimen (N), collected from general anaesthesia and local anaesthesia groups of subjects.

Bacteriome was detected in all OR samples (100%) from both anaesthesia groups. Consistently higher detectable rates were observed in BAL from general (18/28, 64.29%) than local anaesthesia groups (12/31, 38.71%), and in all BAL subgroups (Figure 1D, Supporting Information Figure S1). We saw a slightly lower level of operational taxonomic units (OTUs) in OR, but a higher level in BAL from general than local anaesthesia group (Figure 2A), however, the mouth–lung microbial composition for the top six major genera was almost identical between the two anaesthesia groups, despite the aspiration‐prone nature in patients under general anaesthesia, evidenced by video recorded during bronchoscopy assessment (Supporting Information Video S1). Distinct bacterial composition was outlined (Supporting Information Figure S3) and analysed by Wilcoxon rank‐sum test between OR and BAL (Supporting Information Figure S4), as well as in different subgroups (Supporting Information Figures S5‐S10) under both anaesthesia strategies. The β‐diversity similarity analyses were performed by principal coordinate analysis (PCoA) and non‐metric multidimensional scaling (NMDS), both indicating a separated profile between OR and BAL, but not between the same sites from different anaesthesia groups (Figure 2C,D). Analyses of α‐diversity showed a distinct bacterial community richness, indexed by Ace, Sobs and Chao, between OR and BAL (and subgroups) in the local anaesthesia group, which were completely absent in the general anaesthesia group (Figure 3A). The bacterial community diversity, reflected by Shannon and Simpson indexes, representedno significant difference between OR and BAL (and subgroups) in neither the general nor local anaesthesia groups (Figure 3A). Moreover, Procrustes analysis was performed in the 18 (general anaesthesia) and 12 (local anaesthesia) participants providing both OR and BAL samples, which also indicated a similar undistinguishable profile between OR and BAL in both anaesthesia groups (Figure 3B).

**FIGURE 2:**
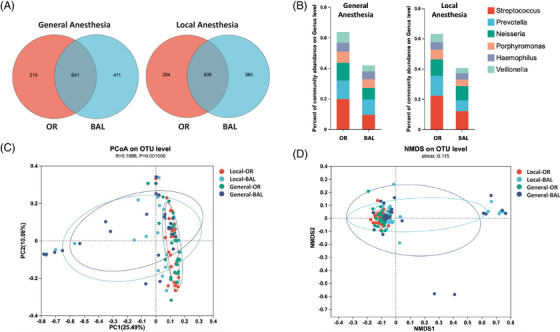
Divergent bacterial profile in OR and BAL samples. (A) Venn diagram showing the shared and unique operational taxonomic units (OTUs) detected in OR and BAL from general and local anaesthesia groups. (B) Bacterial composition in OR and BAL from general and local anaesthesia groups on genus level. (C) Principal coordinate analysis (PCoA) based on Bray‐Curtis Dissimilarity index (PERMANOVA, *p* = 0.001). (D) Non‐metric multidimensional scaling (NMDS, Adonis *p* = 0.001).

**FIGURE 3:**
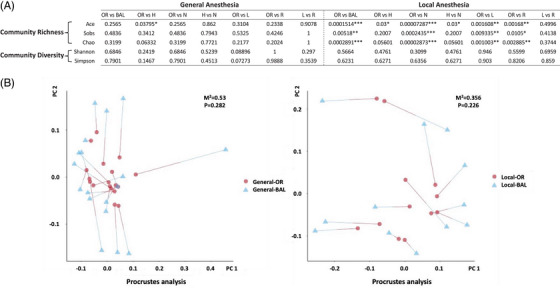
Comparison of OR‐BAL microbial pattern between general and local anaesthesia groups. (A) Table showing indexes for microbial community richness (Ace, Sobs and Chao) and diversity (Shannon and Simpson) in OR and BAL (including subgroups) specimens from general and local anaesthesia groups of subjects. The indexes were calculated and analysed based on OTUs. *p < 0.05, **p < 0.01 and ***p < 0.001. (B) Procrustes analysis showing the similarity and calculated *p* value between OR and BAL microbiome in general and local anaesthesia groups.

To further analyse the community signature in BAL samples, Dirichlet Multinomial Mixtures (DMM) analysis was performed in BAL of the 18 (general anaesthesia) and 12 (local anaesthesia) participants. Three community types were observed with Type 1, representing the majority of the samples (20/30) which indicated a generally unseparated profile between the two anaesthesia groups (Figure 4A,B). Furthermore, unsupervised hierarchical clustering also indicated mixed and non‐separated clusters in OR (Figure 4C) and partially separation in BAL (Figure 4D) of the two anaesthesia groups.

**FIGURE 4:**
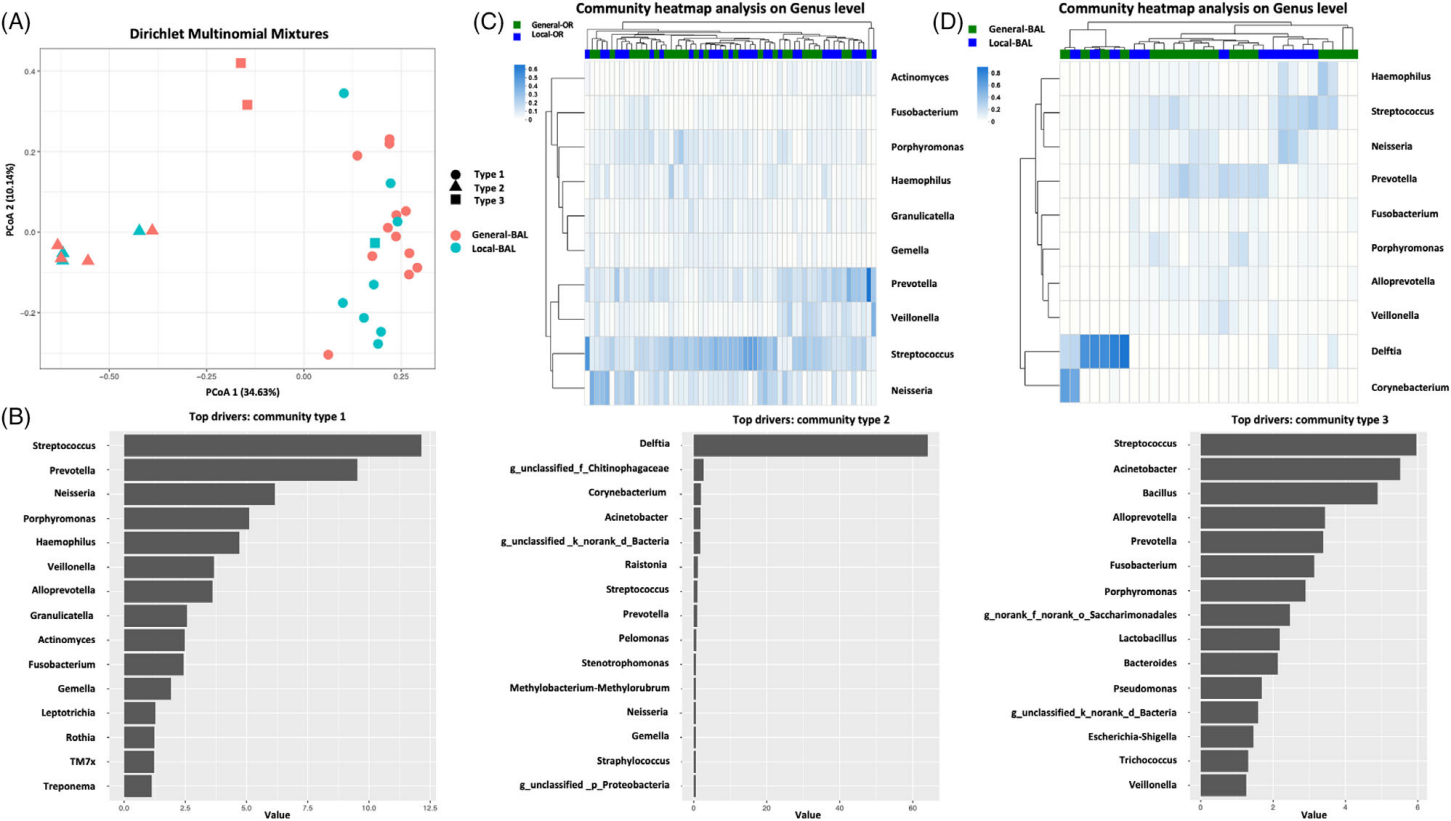
Microbial cluster profile between BAL samples from general and local anaesthesia groups. (A, B) Dirichlet Multinomial Mixtures (DMM) model (A) showing the three different community types (B) in BAL from general and local anaesthesia groups of subjects. (C, D) Heat maps of unsupervised hierarchical clustering of OR (C) and BAL (D) samples. General anaesthesia group was indicated with green labels and local anaesthesia group with blue labels. Dendrogram identified similar major BAL microbiomes as the DMM community types (B).

Oral microbes are not only closely related with oral or dental diseases, but also with lung diseases. The high detection of oral microbes in sputum and BAL specimens implies their association and potential causal relationship with many chronic and infectious lung diseases such as pneumonia, chronic obstructive pulmonary disease, cystic fibrosis, asthma and COVID‐19.^8^ Previous studies reported a high bacterial load of *Prevotella* and *Veillonella* in human BAL,^9^ which was associated with pro‐inflammation phenotype in the lower airway.^10^ Therefore, the higher bacterial load in BAL and other lung specimens could be a long‐term risk that leads to prolonged alteration of lung microbiota composition and provides a potential explanation for the clinical observation of secondary lung infection in post‐bronchoscopy patients. Although this hypothesis is limited by the current short‐term preliminary study based on the slightly increased OTUs and significant microbial richness in BAL from general anaesthesia group, we believe future large‐scale and time‐course cohorts will further evaluate if general anaesthesia could pre‐dispose the mouth–lung microbial immigration and increase the long‐term risk for lung infection and microbial dysbiosis.

## CONCLUSIONS

This study first compared the effects of two commonly used anaesthesia strategies on the lung microecosystem by using bronchoscopy sampling and sequencing. Based on data from the short‐term pilot study, we found that although general anaesthesia slightly enhanced the mouth–lung microbial communication, likely through increased aspiration, similar mouth–lung microbial diversity and composition were observed under both anaesthesia strategies.

## CONFLICT OF INTEREST STATEMENT

The authors declare no conflict of interest.

## FUNDING

This work was supported in part by the grants from the National Natural Science Foundation of China (82170065, 82241012, 82120108001, 81970057 and 82270052), National Key Research and Development Program of China (2022YFE0131500 and 2018YFC1311900), Local Innovative and Research Teams Project of Guangdong Pearl River Talents Program (2017BT01S155), Guangdong Basic and Applied Basic Research Foundation (2021A1515010767 and 2022A1515012564), Guangzhou Basic Research Program Municipal School (Hospital) Joint Funded Foundation and Application Basic Research Project (ZNSA‐2020003), Basic Science and Application of Guangzhou Science and Technology Plan (202201020410, 202201020538 and 202102020019), Independent Project of State Key Laboratory of Respiratory Disease (SKLRD‐Z‐202219), Guangdong‐Hong Kong‐Macao Joint Laboratory of Respiratory Infectious Disease (GHMJLRID‐Z‐202110 and GHMJLRID‐Z‐202120), Open Research Funds from The Sixth Affiliated Hospital of Guangzhou Medical University (Qingyuan People's Hospital) (202201−101 and 202201−309) and Plan on Enhancing Scientific Research in Guangzhou Medical University.

## Supporting information

Supporting information

Supporting information

## Data Availability

All the data supporting the results of the present study are available from the corresponding authors upon reasonable request.
