# Supplementary material for: Impacts of anaesthesia strategies on mouth–lung microbial signature: Evidence from bronchoscopy sampling and sequencing
Source: Clin Transl Med. 2023 Jul 28;13(8):e1355. doi: 10.1002/ctm2.1355 (PMC10382496; doi:10.1002/ctm2.1355)
Supplement: Supplementary file 1 — Supporting information [file CTM2-13-e1355-s002.docx]

Supplementary Material for

Impacts of Anesthesia Strategies on Mouth-Lung Microbial Signature: Evidence from Bronchoscopy Sampling and Sequencing

Chunli Tang^1,#^, Chenting Zhang^1,#^, Qian Jiang^1,#^, Rongmei Geng^1^, Jingnan Zhai^1^,

Jian Wang^1,2,3,*^, Shiyue Li^1,*^, Kai Yang^1,*^

Correspondence to: Kai Yang (kyang28@pku.edu.cn), Shiyue Li (lishiyue@188.com) and Jian Wang (jiw037@health.ucsd.edu)

**This PDF file includes:**

Materials and Methods

Figures S1 to S10

**Materials and Methods**

**Anesthesia strategies**

Patients were inhaled 5 mL of 5% lidocaine (5 min) by nebulization. After nebulization, subjects were randomly assigned into general and local anesthesia groups. In the general anesthesia group, propofol (1.5-2.0 mg/kg) and sufentanil (0.1-0.3 μg/kg) were intravenously given to induce anesthesia and then propofol (4-6 mg/kg/h) was maintained until the completion of procedure, during which the subjects were monitored by the bispectral index BIS (maintained at 80-100). In the local anesthesia group, bronchoscope operation was completed without intravenous anesthetic drugs.

**DNA extraction and PCR amplification**

Microbial DNA was extracted from samples according to the manufacturer’s protocols. The final DNA concentration was determined by NanoDrop 2000 UV-vis spectrophotometer (Thermo Scientific, Wilmington, USA), and the purification quality of DNA was evaluated by 1% agarose gel electrophoresis. The V3-V4 hypervariable regions of the bacteria 16S rRNA gene were amplified with the primer pair: 338F (5’-ACTCCTACGGGAGGCAGCAG-3’) and 806R (5’-GGACTACHVGGGTWTCTAAT-3’) by thermocycler PCR system (ABI GeneAmp 9700, ABI, USA). The PCR reactions were conducted using the following program: 3 min of denaturation at 95 °C, 27 cycles of 30 sec at 95 °C, 30 sec for annealing at 55 °C, and 45 sec for elongation at 72 °C, and a final extension at 72 °C for 10 min. PCR reactions were performed in triplicate 20 μL mixture containing 4 μL of 5×FastPfu Buffer, 2 μL of 2.5 mM dNTPs, 0.8 μL of each primer (5 μM), 0.4 μL of FastPfu Polymerase, 0.2 μL of BSA and 10 ng of template DNA. The yielding PCR products were extracted from a 2% agarose gel and further purified using the AxyPrep DNA Gel Extraction Kit (Axygen Biosciences, Union City, CA, USA) and quantified according to the manufacturer’s protocol. Control samples including Sterile Saline (SS), Brush Saline (BS) and Scope Saline Rinse (SSR) were collected, PCR amplified and sequenced for quality control of OR and BAL samples.

**Illumina MiSeq sequencing** **and data processing**

The purified amplicons were pooled in equimolar and paired-end sequenced (2×300) on an Illumina MiSeq platform (Illumina, San Diego, USA) according to the standard protocols by Majorbio Bio-Pharm Technology Co. Ltd. (Shanghai, China). Quality-controlled and analyzed on the Majorbio Cloud Platform developed and maintained by the Shanghai Majorbio Bio-Pharm Technology Co., Ltd, Shanghai, China (https://cloud.majorbio.com). Raw fasta files were demultiplexed, quality filtered by Trimmomatic and merged by FLASH with the following criteria: *1)* The reads were truncated at any site receiving an average quality score <20 over a 50 bp sliding window. *2)* Primers were exactly matched allowing 2 nucleotide mismatching, and the reads containing ambiguous bases were removed. *3)* The taxonomic distribution of sequences and operational taxonomic units (OTUs) were clustered with 97% similarity cutoff using UPARSE (version 7.1 http://drive5.com/uparse/) and chimeric sequences were identified and removed using UCHIME. The taxonomy of each sequence was analyzed by RDP Classifier algorithm (http://rdp.cme.msu.edu/) against the Silva (SSU123) 16S rRNA database using a confidence threshold of 70%. R package Decontam was used for decontamination by using prevalence-based strategy. In specific, microbial taxa that were highly detected in the sequencing data from the three negative control samples (SS, BS and SSR) were suspected sequences from contamination and removed in the sequencing data from OR and BAL samples.

**Statistical analysis**

Based on the observed OTUs profile, the α-diversity was measured with the indices of Ace, Sobs, Chao, Shannon and Simpson indexes, respectively. The results were displayed in the mothur software (version v.1.30.1). The β-diversity in OR and BAL samples from both anesthesia groups were analyzed and visualized by principal coordinate analysis (PCoA) and non-metric multidimensional scaling (NMDS). Wilcoxon Rank-sum test was employed to analyze the taxonomic difference between sampled sites at genus level. Procrustes analysis was performed by Vegan package of R to analyze the similarity between OR and BAL samples from both anesthesia groups. Dirichlet Multinomial Mixtures (DMM) model was performed by R to analyze the different community types in BAL from general and local anesthesia groups.

**Supplementary Figures**

**Figure S1:** Detailed microbial detection information in oral rinse specimen (OR) and bronchoalveolar lavage specimen (BAL), as well as different BAL subgroups, collected from each individual subject of general anesthesia and local anesthesia groups. “+” indicates positive detection and “–” indicates negative detection (Related to **Fig. 1D**).

**Figure S2:** Agarose electrophoresis data showing the PCR amplified DNA products using 338F (5’-ACTCCTACGGGAGGCAGCAG-3’) and 806R (5’-GGACTACHVGGGTWTCTAAT-3’) (**A**) and extracted bacterial genome DNA (**B**) from samples including Sterile Saline (SS), Brush Saline (BS) and Scope Saline Rinse (SSR), as well as OR and BAL samples as indicated in the note.

**Figure S3:** Microbial profile on genus level in OR and BAL (**A**) and in SS, BS and SSR (**B**) from general and local anesthesia groups. Data were represented as relative abundance (%) in each group.

**Figure S4:** Microbial difference on genus level between OR and BAL in general anesthesia (**A**) and local anesthesia (**B**) groups. Data were represented as relative abundance (%) in each group. Statistical analysis was performed by the Wilcoxon Rank-sum test. **P* < 0.05, ***P* < 0.01 and ****P* < 0.001.

**Figure S5:** Microbial difference on genus level between OR and left lingular segment BAL (L-BAL) in general anesthesia (**A**) and local anesthesia (**B**) groups. Data were represented as relative abundance (%) in each group. Statistical analysis was performed by the Wilcoxon Rank-sum test. **P* < 0.05, ***P* < 0.01 and ****P* < 0.001.

**Figure S6:** Microbial difference on genus level between OR and right middle lung lobe BAL (R-BAL) in general anesthesia (**A**) and local anesthesia (**B**) groups. Data were represented as relative abundance (%) in each group. Statistical analysis was performed by the Wilcoxon Rank-sum test. **P* < 0.05, ***P* < 0.01 and ****P* < 0.001.

**Figure S7:** Microbial difference on genus level between OR and healthy lung lobe BAL (H-BAL) in general anesthesia (**A**) and local anesthesia (**B**) groups. Data were represented as relative abundance (%) in each group. Statistical analysis was performed by the Wilcoxon Rank-sum test. **P* < 0.05, ***P* < 0.01 and ****P* < 0.001.

**Figure S8:** Microbial difference on genus level between OR and nodular lung lobe BAL (N-BAL) in general anesthesia (**A**) and local anesthesia (**B**) groups. Data were represented as relative abundance (%) in each group. Statistical analysis was performed by the Wilcoxon Rank-sum test. **P* < 0.05, ***P* < 0.01 and ****P* < 0.001.

**Figure S9:** Microbial difference on genus level between L-BAL and R-BAL in general anesthesia (**A**) and local anesthesia (**B**) groups. Data were represented as relative abundance (%) in each group. Statistical analysis was performed by the Wilcoxon Rank-sum test. **P* < 0.05, ***P* < 0.01 and ****P* < 0.001.

**Figure S10:** Microbial difference on genus level between H-BAL and N-BAL in general anesthesia (**A**) and local anesthesia (**B**) groups. Data were represented as relative abundance (%) in each group. Statistical analysis was performed by the Wilcoxon Rank-sum test. **P* < 0.05, ***P* < 0.01 and ****P* < 0.001.
